# Inferring time series chromatin states for promoter-enhancer pairs based on Hi-C data

**DOI:** 10.1186/s12864-021-07373-z

**Published:** 2021-01-28

**Authors:** Henriette Miko, Yunjiang Qiu, Bjoern Gaertner, Maike Sander, Uwe Ohler

**Affiliations:** 1 Berlin Institute for Medical Systems Biology, Max Delbrück Center for Molecular Medicine, 13125 Berlin, Germany; 2 Department of Computer Science, Humboldt-Universität zu Berlin, 10117 Berlin, Germany; 3 Ludwig Institute for Cancer Research, La Jolla, CA 92093 USA; 4 Bioinformatics and Systems Biology Graduate Program, University of California San Diego, La Jolla, CA 92093 USA; 5 Department of Cellular and Molecular Medicine, University of California San Diego, La Jolla, CA 92093 USA; 6 Department of Pediatrics, Pediatric Diabetes Research Center, University of California San Diego, La Jolla, CA 92093 USA; 7 Department of Biology, Humboldt-Universität zu Berlin, 10117 Berlin, Germany

**Keywords:** Gene regulation, Chromatin immunoprecipitation, Histone modifications, Hi-C, Enhancer, Differentiation

## Abstract

**Background:**

Co-localized combinations of histone modifications (“chromatin states”) have been shown to correlate with promoter and enhancer activity. Changes in chromatin states over multiple time points (“chromatin state trajectories”) have previously been analyzed at promoter and enhancers separately. With the advent of time series Hi-C data it is now possible to connect promoters and enhancers and to analyze chromatin state trajectories at promoter-enhancer pairs.

**Results:**

We present TimelessFlex, a framework for investigating chromatin state trajectories at promoters and enhancers and at promoter-enhancer pairs based on Hi-C information. TimelessFlex extends our previous approach Timeless, a Bayesian network for clustering multiple histone modification data sets at promoter and enhancer feature regions. We utilize time series ATAC-seq data measuring open chromatin to define promoters and enhancer candidates. We developed an expectation-maximization algorithm to assign promoters and enhancers to each other based on Hi-C interactions and jointly cluster their feature regions into paired chromatin state trajectories.

We find jointly clustered promoter-enhancer pairs showing the same activation patterns on both sides but with a stronger trend at the enhancer side. While the promoter side remains accessible across the time series, the enhancer side becomes dynamically more open towards the gene activation time point. Promoter cluster patterns show strong correlations with gene expression signals, whereas Hi-C signals get only slightly stronger towards activation.

The code of the framework is available at https://github.com/henriettemiko/TimelessFlex.

**Conclusions:**

TimelessFlex clusters time series histone modifications at promoter-enhancer pairs based on Hi-C and it can identify distinct chromatin states at promoter and enhancer feature regions and their changes over time.

**Supplementary Information:**

The online version contains supplementary material available at 10.1186/s12864-021-07373-z.

## Background

Genomic regulatory regions like promoters and enhancers are important players in gene expression. Their activity has been shown to correlate with specific co-localized combinations of post-translational histone modifications (or marks) called ”chromatin states”. For example, active promoters are enriched in histone modifications H3 lysine 27 acetylation (H3K27ac) and H3 lysine 4 di−/trimethylation (H3K4me2/3), while active enhancers are enriched in H3K27ac and histone H3 lysine 4 mono−/dimethylation (H3K4me1/2). Whether histone modifications are causal or a consequence of the activity of the genomic locus remains unclear.

Chromatin states have initially been annotated in a spatial manner genome-wide, by segmenting the genome into distinct states based on histone modification ChIP-seq data from, for instance, one cell line, which represents an unsupervised learning problem. Chromatin states were popular in the Encyclopedia of DNA Elements (ENCODE) [1], resulting from the first seminal methods ChromHMM [2] and Segway [3]. In ChromHMM, the genome is partitioned into 200 bp bins, and a multivariate Hidden Markov Model (HMM) with binary values represented as Bernoulli random variables is used to model the combinatorial presence or absence of histone marks in all bins [2]. In Segway, a Dynamic Bayesian Network modelling the read counts as independent Gaussian random variables is used to segment and label the genome at base-pair resolution into joint histone mark patterns [3]. Segway was later extended by a graph-based regularization method for incorporating chromatin interaction data from Hi-C, which showed improved results [4]. Other methods for segmentation of a genome include jMOSAiCS [5], EpiCSeg [6] and Spectacle [7].

Several methods focusing on regulatory regions have been introduced, for example over multiple human cell lines [8, 9], using self-organizing maps [10], employing Hi-C data [11, 12], as well as our own approach employing an HMM for chromatin states at high resolution [13].

With the advent of new genomics technologies and improved biological in vitro differentiation systems, time series ChIP-seq data sets have been generated that allow for investigating chromatin states across multiple time points. Such sequential chromatin states are referred to as ”chromatin state trajectories”, and only a handful of methods have been developed to analyze these.

An early method for analyzing chromatin state trajectories is GATE [14], which clusters multiple histone modifications over multiple time points with a hierarchical probabilistic model. The top layer consists of a finite mixture model for clustering genomic segments, and the bottom layer models the temporal changes as an HMM with the two states active and inactive. The limitations of GATE are that it can only handle two states (active/inactive), and that it is not possible to use it on differentiation with more complex topologies. A newer method is CMINT [15], a probabilistic clustering approach to identify chromatin states across multiple cell types, based on a given tree topology representing the relationship of these cell types as input. A limitation of this method is that it uses large genomic regions of 2 or 8 kb. Further methods based on similar ideas include TreeHMM [16] and ChromstaR [17]. Interesting research questions that could be addressed with such methods are: which chromatin states occur during differentiation and how do they change over time? Which genes and enhancers function at specific time points? What are the target genes of these enhancers?

These existing methods generally investigate chromatin states at promoters and enhancers separately. Chromatin interaction data like Hi-C should in principle enable an assignment of promoters and enhancers to promoter-enhancer pairs. Following this idea, we here present TimelessFlex, a model for investigating chromatin state trajectories at feature regions around promoters and enhancers and at pairs of such feature regions. TimelessFlex employs our previous model Timeless [18], a Bayesian network for co-clustering multiple time series histone modifications at given feature regions, which assigns the regions to the cluster with the highest probability. The output are clusters of regions with similar chromatin state trajectories. We extend this approach by (1) a strategy to employ time series ATAC-seq data to improve definitions of promoters and distal regions called ”enhancer candidates”; (2) an expectation-maximization (EM) based approach to allow the use of incomplete or low-resolution time series Hi-C data indicating chromatin interactions; (3) jointly clustering paired chromatin state trajectories; for (4) linear and tree-shaped differentation topologies. We validate our approach and the resulting candidate enhancers for the presence of predicted or in vivo occupied transcription factor (TF) binding sites, for discovering new enhancers, and for linking enhancers to their target genes.

## Results

We developed a Bayesian network-based clustering approach to characterize regulatory regions based on their chromatin state changes across time. A set of candidate regulatory regions is first annotated from ATAC-seq data across the time series. Then, multivariate, quantitative time series histone modification data is used as features for time series clustering, where available Hi-C data allows for the clustering of interacting pairs instead of individual regions. To utilize Hi-C data despite its frequently coarse resolution, we follow a two-step strategy, in which clusters are first determined on unambiguous assignments and in a second round extended by ambiguous interactions, which are resolved via expectation-maximization (EM). As we utilize ATAC-seq and Hi-C merely to define regions and their interactions, but do not exploit the temporal or quantitative information present in ATAC-seq or Hi-C, we also use these data for corroboration.

### Chromatin state trajectories for enhancer feature regions during mouse hematopoiesis

**Fig. 1:**
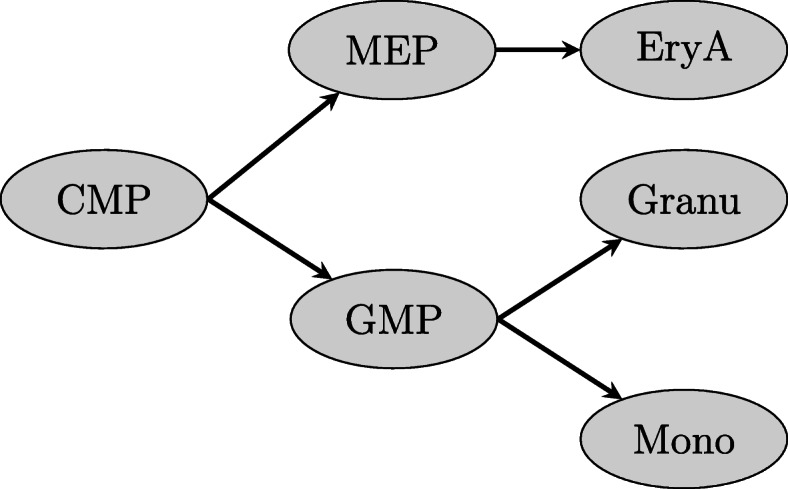
Schematic of mouse hematopoietic differentiation. Six time points of mouse hematopoiesis: common myeloid progenitor (CMP), megakaryocyte erythroid progenitor (MEP), granulocyte macrophage progenitor (GMP), erythrocyte A (EryA), granulocyte (Granu), monocyte (Mono) [19]

**Fig. 2:**
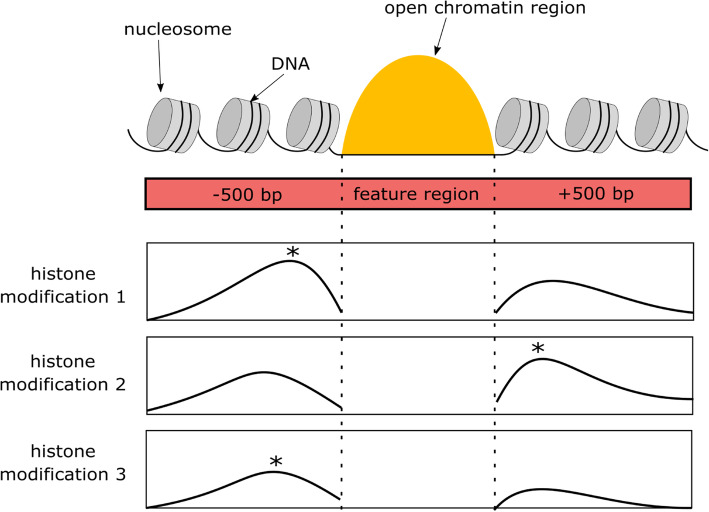
Toy example of a feature region and histone mark signals over it. Top: A feature region (red) is defined as a window around an open chromatin region with 500 bp extension from the edges. Bottom: Three histone modification signals over the feature region are shown. For each histone modification, the maximum signal (*) is computed

We first illustrate the TimelessFlex principles on a data set from mouse hematopoiesis [19] based on a given branching trajectory of differentiation (see Fig. 1), for the scenario that there are time series ChIP-seq and ATAC-seq data available but no accompanying Hi-C data set. We defined one consistent set of distal regions (“enhancer candidates”) across the time series based on ATAC-seq data (see Methods), which resulted in 48,804 enhancer feature regions. As feature region we took the window around an open chromatin region with 500 bp extension from the edges (see Fig. 2, top). To determine an appropriate number of clusters, Akaike information criterion (AIC) and Bayesian information criterion (BIC) were computed and clusters corresponding to local minima were visually inspected. This led to 19 clusters of enhancer regions (see Additional file 1: Figure S1 for model selection and Additional file 2: Figure S2 for all 19 enhancer clusters).

**Fig. 3:**
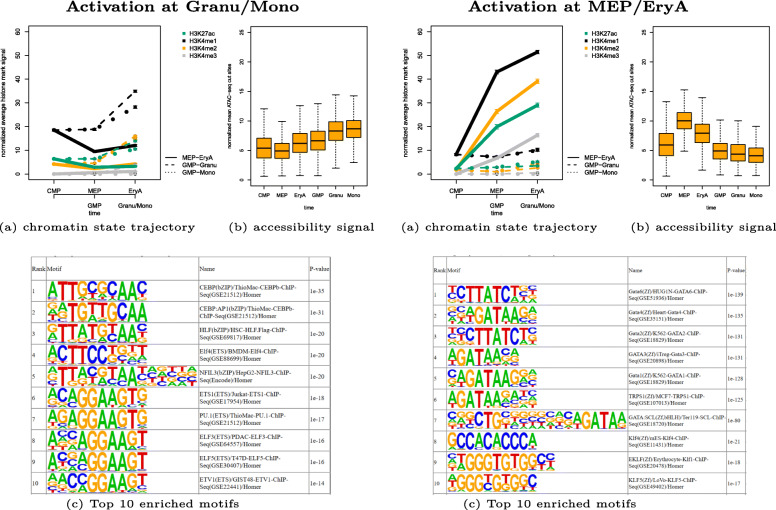
Example clusters of enhancer feature regions during mouse hematopoiesis. Left: activation at Granu/Mono (cluster 11 with 2480 feature regions), right: activation at MEP/EryA (cluster 7 with 983 feature regions), **a** shows chromatin state trajectory, **b** accessibility signal from ATAC-seq, **c** Top 10 known enriched motifs by HOMER

Figure 3 illustrates the impact of chromatin state clustering across time and different lineages simultaneously, for two example clusters of enhancer feature regions. Cluster 11 consists of 2480 regions that become more active at time points granulocyte (Granu) and monocyte (Mono). The corresponding ATAC-seq signal confirms that the enhancer regions are more accessible at these stages compared to other time points. Enriched transcription factor motifs computed with HOMER come from the CEBP family and PU.1. Cebpb, Cebpa and PU.1 are known regulators of myeloid enhancers and Cebpb was shown to be an important TF for lineage specification of granulocytes [19]. Cluster 7 with 983 enhancer feature regions becomes active towards the MEP and EryA stages. At these time points the ATAC-seq signal shows a strong increase in accessibility. HOMER found enriched motifs for Gata, GATA binding TF TRPS1 and Klf families, where Gata1 and Klf1 in particular are known regulators of erythroid enhancers [19].

### Chromatin state trajectories during human pancreatic differentiation

**Fig. 4:**
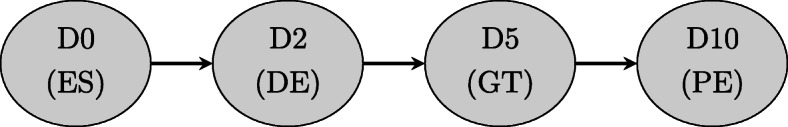
Schematic of human pancreatic differentiation system. Four time points of human pancreatic differentiation: day 0 (D0) human embryonic stem cells (ES cells), day 2 (D2) definitive endoderm (DE), day 5 (D5) primitive gut tube (GT), day 10 (D10) pancreatic endoderm (PE) [20, 21]

The main application of TimelessFlex addresses an extensive multi-omics time series data set, including deep Hi-C data, obtained at multiple stages of human pancreas differentiation (see Fig. 4).

#### Chromatin state trajectories for enhancer feature regions

**Fig. 5:**
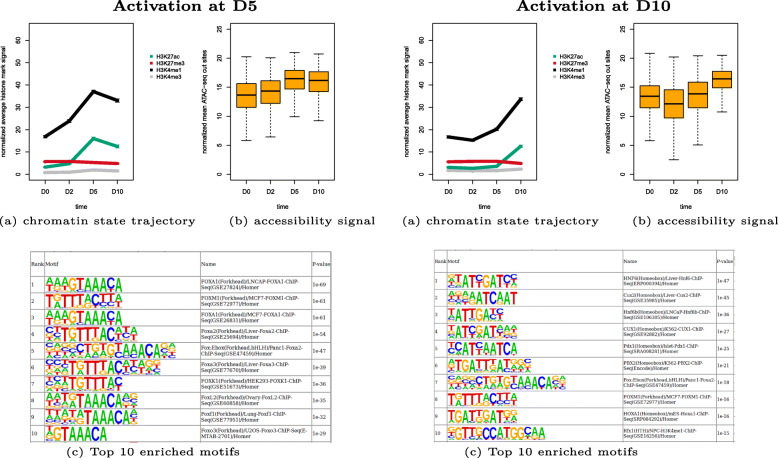
Example clusters of enhancer feature regions during human pancreatic differentiation. Left: activation at D5 (cluster 6 with 1431 feature regions), right: activation at D10 (cluster 5 with 1451 feature regions), **a** shows chromatin state trajectory, **b** accessibility signal from ATAC-seq, **c** Top 10 known enriched motifs by HOMER

As in the case of hematopoiesis above, we started by annotating enhancer feature regions from ATAC-seq data. We obtained 17,103 enhancer feature regions and clustered them in 8 clusters (see Additional file 3: Figure S3 for model selection and Additional file 4: Figure S4 for all 8 clusters). As examples, Fig. 5 shows details for cluster 6 (active at D5) and cluster 5 (active at D10). Cluster 6 consists of 1431 enhancer feature regions that show strong activity at D5 and decreased activity at D10. The regions become more open at D5 and slightly less open at D10. HOMER results show motifs for the FOX family. Cluster 5 with 1451 feature regions becomes active at D10 and the features regions become more open towards D10. HOMER reported motifs for HNF, CUX, Pdx1, PBX1 and FOX family.

#### Paired chromatin state trajectories for promoter-enhancer pairs

The multi-stage Hi-C data allowed for a joint characterization of interacting promoters and enhancers. Promoter-enhancer candidate pairs were determined based on ATAC-seq and Hi-C data (see Methods) and led to 3617 initialization feature pairs and 3406 multi feature pairs. This illustrates the main motivation behind our semi-supervised approach, namely that the current Hi-C coverage and resolution frequently does not enable an unambiguous assignment between all promoters and enhancers.

##### Initialization feature pairs

**Fig. 6:**
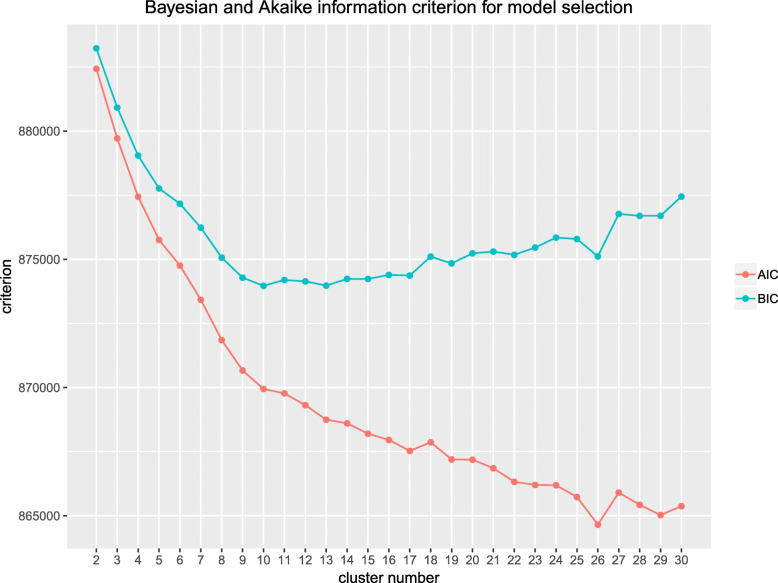
Model selection for clustering of promoter-enhancer initialization feature pairs during human pancreatic differentiation. Bayesian information criterion (BIC) and Akaike information criterion (AIC) are computed in the range of 2 to 30 clusters to decide on the number of clusters for the initialization feature pairs. Cluster number 10 is the minimum of the BIC in the investigated range and therefore chosen as cluster number

For clustering the initialization feature pairs, 10 clusters were determined as the optimal BIC in the investigated range (Fig. 6). All 10 initialization clusters can be found in Additional file 5: Figure S5.

**Fig. 7:**
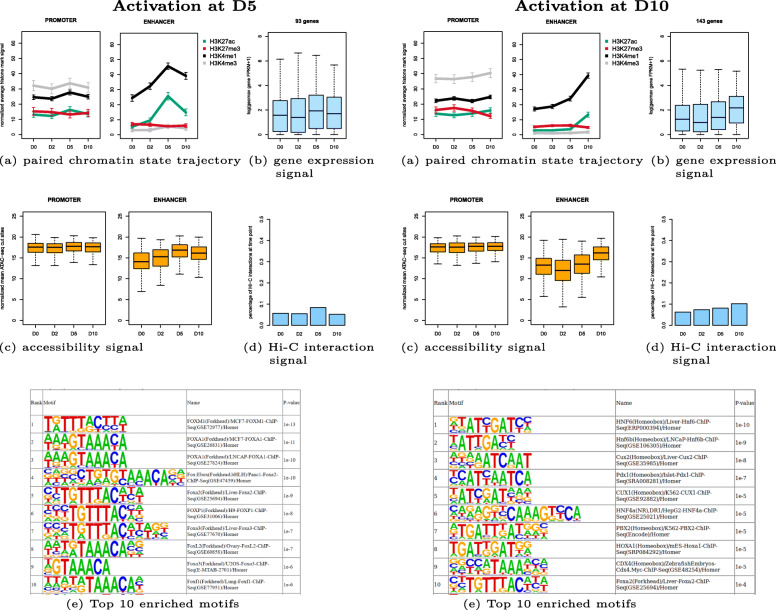
Example clusters of initialization promoter-enhancer feature pairs during human pancreatic differentiation. Left: activation at D5 (cluster 7 with 226 initialization feature pairs), right: activation at D10 (cluster 3 with 282 initialization feature pairs), **a** shows paired chromatin state trajectory, **b** gene expression signal from RNA-seq, **c** accessibility signal from ATAC-seq, **d** interaction signal from Hi-C, **e** Top 10 known enriched motifs by HOMER

**Fig. 8:**
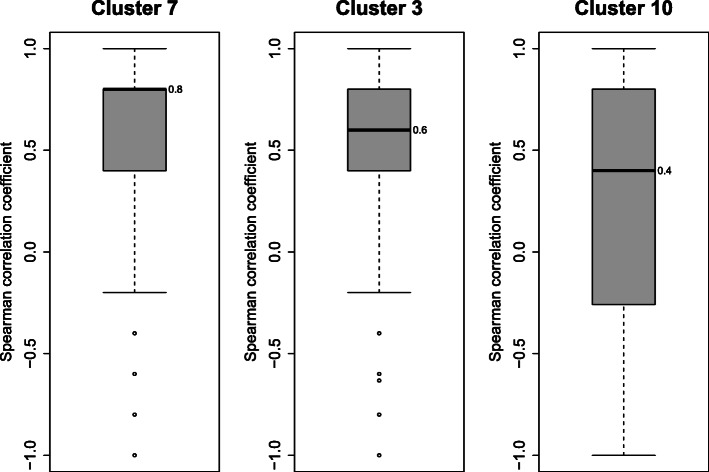
Spearman correlation of H3K27ac signal and ATAC-seq signal for enhancer clusters. For clusters 7, 3 and noise cluster 10 the Spearman correlation coefficient was computed between H3K27ac signal and ATAC-seq signal for each feature region. For clusters 7 and 3 the correlation is higher than for the noise cluster 10

**Fig. 9:**
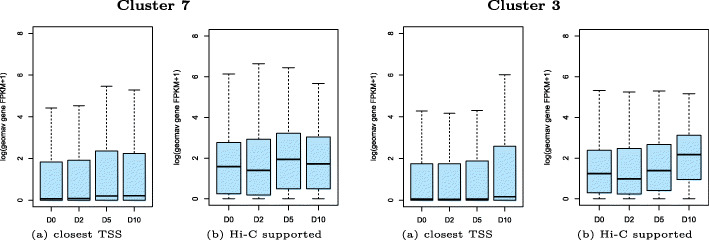
Comparison of gene expression signals for closest TSS and Hi-C supported genes. Gene expression signal from RNA-seq for (**a**) genes with closest TSSs to enhancers in cluster 7 and 3 from initialization pairs and for (**b**) Hi-C supported assigned genes

Two example clusters are shown in Fig. 7: cluster 7 with pairs becoming active at time point D5 and cluster 3 with pairs becoming active at D10. To evaluate the success of the unsupervised clustering, we aimed to assess the quality of cluster membership in different ways. For one such metric we used the quantitative ATAC-seq signal which is not used for clustering. More precisely, we computed the Spearman correlation co-efficent between H3K27ac signal and ATAC-seq signal for each enhancer feature region in clusters. For cluster 7, the median correlation coefficient is 0.8, and for cluster 3 it is 0.6 (Fig. 8). The correlation of the noise cluster is 0.4 and served as adequate baseline. In addition to the higher median correlation, the distributions of the correlation coefficients in clusters 7 and 3 are also much narrower. As another measure, we computed the RNA-seq derived gene expression levels of the closest transcript TSSs as baseline, to compare them to the Hi-C supported assignments. Figure 9 shows a much weaker gene expression of the baseline assignments compared to the cluster-assigned promoters in Fig. 7 (see Additional file 6: Figure S6 for all clusters).

Cluster 7 (Fig. 7, left side) consists of 226 promoter-enhancer pairs. The paired chromatin state trajectory shows that the enhancers get activated strongly at D5 and then lose their signal at D10. The promoters exhibit the same trajectory but much weaker, in accordance with reports that documented the much lower variability in the accessibility of promoters, which are frequently open even if the genes are not actively transcribed [22]. When looking at the gene expression signal from the RNA-seq, it confirms that steady-state gene expression is elevated at D5. The Hi-C signal confirms that the highest number of interactions is observed at D5, but some interactions persist at other days. Given that we are only analyzing a subset of active regions, we observed small overlaps with reported signature genes for different stages (1/90 at D2, 1/18 at D5, 1/31 at D10). Motif analysis of the enhancer candidates with HOMER found motifs from the FOX family.

In cluster 3 (displayed in Fig. 7, right side) there are 282 promoter-enhancer pairs. The enhancers get strongly activated at D10, while the promoters show a weaker increase at D10. The gene expression signal gets increased at D10, and the Hi-C signal again shows the highest number of interactions at D10. For this cluster, there is a clear enrichment for known signature genes from D10 (3/90 at D2, 0/18 at D5, 14/31 at D10). Motifs of HNF and CUX families, Pdx1 and PBX2 were found by HOMER as enriched in enhancer regions.

Pairwise intersections of enhancers from cluster 7 and cluster 3 with published FOXA1, FOXA2 and PDX1 ChIP-seq peaks and Fisher’s test showed a highly significant overlap of FOXA ChIP targets in cluster 3 and of PDX1 in cluster 7, respectively (Table 1). As both clusters contain genes active in pancreatic differentiation, TF interactions were generally enriched in both clusters, but the most significant enrichment was observed for D5 for cluster 7 and FOXA1/2, i. e. at the point of highest enhancer activation, and for D10 for cluster 3 in the case of PDX1.

**Table 1:** Results of Fisher’s two-tailed test. Test performed on pairwise intersections of enhancer clusters from initialization pairs with published ChIP-seq peaks [20]

ChIP-seq peaks	cluster 7	cluster 3
FOXA1 at D5	**5.3257e-102**	6.4588e-22
FOXA1 at D10	1.5073e-31	2.0066e-87
FOXA2 at D2	6.0772e-115	6.3536e-19
FOXA2 at D5	**7.0324e-180**	9.476e-41
FOXA2 at D10	2.5729e-123	5.7507e-81
PDX1 at D10	1.0513e-54	**2.1041e-132**

Altogether, this demonstrates that our approach can (a) identify distinct chromatin trajectories which are (b) supported by complementary genomics data, are (c) enriched in sequence motifs and functional interactions of known relevant TFs, and (d) enrich for enhancers with an impact on gene expression compared to the baseline of the closest assignment. Our observations also support the current understanding that histone modifications and chromatin accessibility is much more pronounced at individual enhancers, rather than the promoters that act as integration platforms of multiple regulatory regions.

##### Multi feature pairs

While the pancreas lineage Hi-C data is of very high depth, it still allowed for an unambiguous assignment of only ∼3600 enhancers. Given that clustering is based on a probabilistic graphical model, we wondered whether it would be possible to not only use it to infer unobservable cluster identities, but also resolve multi pair regions. In such regions Hi-C shows interactions between regions with multiple enhancers and/or promoters. Our data set consists of almost as many multi pairs as unambiguous pairs.

**Fig. 10:**
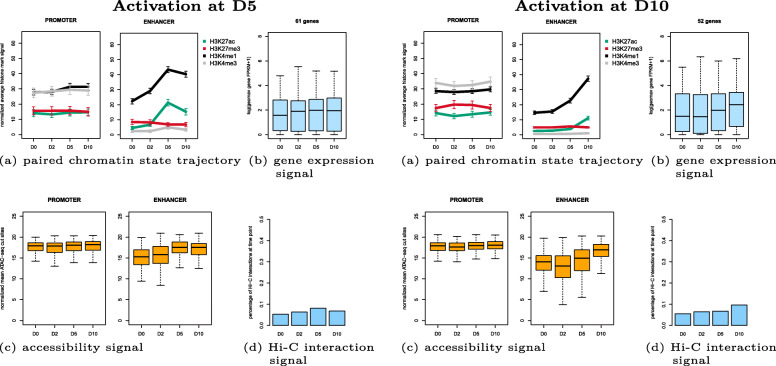
Example clusters of multi promoter-enhancer feature pairs during human pancreatic differentiation. Only the selected multi regions are plotted. Left: activation at D5 (cluster 7 with 225 multi feature pairs), right: activation at D10 (cluster 3 with 255 multi feature pairs), **a** shows paired chromatin state trajectory, **b** gene expression signal from RNA-seq, **c** accessibility signal from ATAC-seq, **d** interaction signal from Hi-C

These multi feature pairs were thus clustered in a second step, using the model resulting from clustering the initialization pairs. The cluster number and the cluster ordering stayed fixed (e. g. cluster 7 stays cluster 7 for ambiguous pairs; see Additional file 7: Figure S7 for all 10 multi clusters). 753 of 3406 ambiguous pairs were assigned to the noise cluster. The newly determined promoter-enhancers from this larger set of pairs are shown in Fig. 10 for cluster 7 and cluster 3. It can be seen that the ambiguous pair clusters are very similar to their corresponding initialization clusters, and are equally well supported by RNA-seq, ATAC-seq, and Hi-C data.

In summary, our EM based assignment of ambiguous Hi-C interactions nearly doubled the number of assignments of promoters to enhancers, while the agreement with orthogonal functional genomics data was on par with the unambiguous pairs. This suggests that the activity of these enhancers has an equal impact on gene expression as those used for initial clustering, but that the genomic arrangement and spatial resolution did not allow them to be directly assigned.

## Discussion

TimelessFlex learns chromatin state trajectories of promoter and enhancer feature regions and of promoter-enhancer feature pairs during differentiation by co-clustering multiple histone modification data sets. It identifies clusters of genes that may function at specific stages during differentiation and groups of enhancers that are active at certain time points. Clustering of feature regions of promoter-enhancer pairs, we find clusters where promoters and enhancers show the same activation patterns. Noticeably, the trend of the histone mark signals of the enhancer side is much stronger compared to the promoter side. We identify enhancer clusters that become active or repressed for nearly every stage of two example differentation data sets from hematopoiesis and pancreas development, whereas this is not necessarily the case for promoter clusters. However, as readout of the promoters, the gene expression signal from RNA-seq correlates well with the inferred chromatin trajectories. On the enhancer side, motif enrichment analyses with HOMER reveal known hematopoietic respectively pancreatic and hepatic TFs in active enhancer clusters at specific time points.

Paired clustering allows for direct comparison of the accessibility signals of the promoter and the enhancer. It can be seen that the promoters are near-constantly open across time, while enhancers open more dynamically towards the time point of highest gene activation. Enhancers change in terms of accessibility much more across time, and this correlates with active histone modifications. This suggests that the activity of the promoter is comparatively better predicted by using histone mark signals than accessibility. Looking at Hi-C interactions within clusters, we found that some interactions are observed at each time point, but that their number is highest at the time point of highest activation. This suggests that at least some promoter-enhancer interactions are established long before activation of their target gene.

In the initialization clusters there are 512 promoters and 242 enhancer candidates that were also found in at least one other cluster. Investigation of these feature regions would be an interesting point for future analysis.

We found that resulting chromatin state trajectories from multi clusters are very similar to the clusters obtained from clustering the initialization pairs, indicating that we successfully identified additional promoter-enhancer pairs of equal quality, nearly double the cluster sizes by adding the corresponding multi pairs. To the best of our knowledge, paired chromatin state trajectories have not yet been investigated, which makes it difficult to directly compare to methods for chromatin state analysis such as those discussed in the introduction.

In many studies, feature regions for promoters are commonly defined as regions of fixed lengths around all annotated/expressed genes, e. g. +2 kb and -200 bp around TSSs in our previous approach Timeless [18]. Instead, we here use a data driven approach employing ATAC-seq data for defining precise coordinates of promoter and enhancer candidate regions. A similar strategy was employed in fly in [23], where DHSs from DNase-seq were used as proxy for putative enhancers, reducing the search space to 6*.*4% of the genome. The ATAC-seq data defined open chromatin regions across all time points are of variable sizes, and we chose windows extending the edges of open chromatin regions by 500 bp, which leads to more pronounced histone mark signals compared to fixed-size windows. The categorization of open chromatin regions into promoters and enhancers is based on gene TSSs from GENCODE. Some regions labelled as enhancer candidates may thus be promoters of incompletely annotated lncRNAs. In the last years, it has been shown that separation of promoters and enhancers is not as clear as their original definition suggested, as many promoters display enhancer-like activity [24]. Further work may therefore completely drop the labeling of promoters and enhancers and characterize all Hi-C interacting pairs. As an alternative to infer significant Hi-C interactions the use of GOTHiC [25] could be investigated.

## Conclusions

We present the flexible framework TimelessFlex, which clusters time series histone modifications at promoters and enhancers and at promoter-enhancer pairs. TimelessFlex identifies distinct chromatin states that occur at promoter and enhancer feature regions over a time series such as in vitro differentiation from stem cells, and how they change over time. It can identify groups of genes and enhancers that are active or repressed at specific time points. ATAC-seq is utilized to define promoters and enhancers and Hi-C data is used to assign them to Hi-C interaction pairs. Feature regions of such interaction pairs are jointly clustered into paired chromatin state trajectories, which allows for exploring their 3D relationship over time.

TimelessFlex is applicable to branched trajectories as well and it can be employed to enable a comparison of chromatin state trajectories between two lineages and to identify genes and enhancers that are active in one lineage and inactive in the other.

Finally, the resulting clusters can be further validated and benchmarked with predictive approaches such as deep learning. Neural networks, similar to [26], can be used to find the most predictive sequence features of each cluster via classification. These sequence features can then be used to distinguish different classes of promoters and enhancers, and to ultimately characterize the impact of non-coding sequence variants obtained from GWAS studies or eQTL mappings, e. g [27].

## Methods

### Data sets and data processing

To showcase our approach, we use multiple data sets from two differentiation systems: hematopoietic differentiation in mouse [19] and an in vitro model system of human pancreatic differentiation [20, 21] (see Figs. 1 and 4, respectively).

For mouse hematopoiesis, we downloaded ChIP-seq and ATAC-seq data from GEO under accession number GSE59636 [19]. We employed ChIP-seq data for H3K4me1/2/3 and H3K27ac and ATAC-seq on the following six time points forming a branching tree: common myeloid progenitor (CMP), megakaryocyte erythroid progenitor (MEP), erythrocyte A (EryA), granulocyte macrophage progenitor (GMP), granulocyte (Granu) and monocyte (Mono).

For human pancreatic differentiation we used multiple data sets (ChIP-seq for H3K27ac, H3K27me3 and H3K4me1/3, ATAC-seq, RNA-seq and Hi-C) at four time points: human embryonic stem cells (ES cells) at day 0 (D0), definitive endoderm (DE) at day 2 (D2), primitive gut tube (GT) at day 5 (D5), and pancreatic endoderm (PE) at day 10 (D10). ChIP-seq data for H3K27ac and H3K4me1 were downloaded from GEO under accession number GSE54471 [20], ChIP-seq data for H3K27me3 and H3K4me3 were downloaded from Array Express under accession number E-MTAB-1086 [21] and from GEO under accession number GSE149148 [28]. ATAC-seq data were generated and deposited in GEO under accession number GSE151769. RNA-seq data were downloaded at Array Express under accession number E-MTAB-1086 [21]. In situ Hi-C data for all four time points were downloaded from the 4D Nucleome Data Portal [29] under accession numbers 4DNESOLVRKBM, 4DNESOL9JVE2, 4DNESV11RYSF and 4DNESSSDVO27.

Table 2 gives an overview of the data samples for the different genomic data types.

**Table 2:** Data samples from mouse hematopoietic differentiation [19] (top) and human pancreatic differentiation [20, 21] (bottom). For each genomic data type and each time point the number of replicates is given

**Data type**	**CMP**	**MEP**	**EryA**	**GMP**	**Granu**	**Mono**
H3K27ac	4	1	2	2	2	4
H3K4me1	3	1	2	2	3	4
H3K4me2	3	2	1	3	2	3
H3K4me3	4	2	1	3	2	4
ATAC-seq	3	3	1	2	2	1
**Data type**	**D0**	**D2**	**D5**	**D10**		
H3K27ac	2	2	2	2		
H3K27me3	2	2	2	2		
H3K4me1	2	2	2	2		
H3K4me3	2	2	2	2		
Input	2	2	2	2		
ATAC-seq	2	2	2	2		
RNA-seq	3	3	3	3		
Hi-C	2	2	2	2		

#### ChIP-seq data

All ChIP-seq data samples were processed as follows: Illumina universal adapters were trimmed from reads with Trim Galore 0.6.1 [30] and reads were mapped with Bowtie2 2.3.4.3 [31] to reference genome mm10 for mouse data or hg19/GRCh37 for human data. An in-house script was used to filter for uniquely mapped reads with at most 2 mismatches (similar to [18]). Duplicate reads were removed with samtools 1.9 [32] and sam format was converted to bed format with bed tools 2.27.1 [33].

Peaks for H3K27ac, H3K27me3, H3K4me1 and H3K4me3 were called with JAMM 1.0.7.5 [34] in window mode with fixed bin size 150 and auto filtering of peaks. As there is no Input data available for mouse hematopoiesis data we used data from time point of long-term hematopoietic stem cells (LT-HSC) as control for peak calling. During peak calling the fragment lengths were computed which were needed later for running JAMM Signal Generator.

#### ATAC-seq data

##### Library preparation

ATAC-seq for human pancreatic differentiation [35] was performed on approximately 50,000 nuclei. The samples were permeabilized in cold permeabilization buffer (0.2% IGEPAL-CA630 (I8896, Sigma), 1 mM DTT (D9779, Sigma), Protease inhibitor (05056489001, Roche), 5% BSA (A7906, Sigma) in PBS (10010–23, Thermo Fisher Scientific) for 10 min on the rotator in the cold room and centrifuged for 5 min at 500 x g at 4 °C. The pellet was resuspended in cold tagmentation buffer (33 mM Tris-acetate (pH = 7.8) (BP-152, Thermo Fisher Scientific), 66 mM K-acetate (P5708, Sigma), 11 mM Mg-acetate (M2545, Sigma), 16% DMF (DX1730, EMD Millipore) in Molecular biology water (46000-CM, Corning)) and incubated with tagmentation enzyme (FC-121-1030; Illumina) at 37 °C for 30 min with shaking at 500 rpm. The tagmented DNA was purified using MinElute PCR purification kit (28,004, QIAGEN). Libraries were amplified using NEBNext High-Fidelity 2X PCR Master Mix (M0541, NEB) with primer extension at 72 °C for 5 min, denaturation at 98 °C for 30 s, followed by 8 cycles of denaturation at 98 °C for 10 s, annealing at 63 °C for 30 s and extension at 72 °C for 60 s. After the purification of amplified libraries using MinElute PCR purification kit (28,004, QIAGEN), double size selection was performed using SPRIselect bead (B23317, Beckman Coulter) with 0.55X beads and 1.5X to sample volume. Finally, libraries were sequenced on HiSeq4000 (Paired-end 50 cycles, Illumina).

##### Data processing

Paired-end ATAC-seq data from pancreatic differentiation was processed similarly to [36]: Nextera adapters were trimmed from reads with Trim Galore 0.6.1 and parameter --paired. Reads were mapped with Bowtie2 2.3.4.3 to hg19 reference genome with maximum fragment length of 2000 bp and parameters --no-disconcordant and --no-mixed. Mapped reads were filtered by mapping quality of 20. Duplicates were removed with Picard 2.10.3 [37] Sort-Sam and MarkDuplicates. Sam format was converted to bedpe format with bedtools 2.27.1. An in-house script was used to filter out reads from chromosome M, unplaced and unlocalized scaffolds or alternative haplotypes. To account for the size of the transposase, read pairs were filtered to have a distance of at least 38 bp between them. Finally, reads were cut to their 5′ ends and converted to bed format. Peaks were called with MACS2 2.1.1.20160309 [38] and the Irreproducible Discovery Rate (IDR) framework (2012 version) [39] as follows: peaks were called with MACS2 and parameters --no-model, --shift 100, --extsize 200, --keep-dup all and p-value -p 0.05 for pooled replicates and for each replicate separately. For each replicate the top 100,000 peaks were taken and the script IDR batch-consistency-analysis was run. An IDR threshold of 0.01 was used to select the top peaks from the pooled peak file which were then taken as final peaks.

For single-end ATAC-seq data from hematopoiesis, Nextera adapters were trimmed from reads with Trim Galore 0.6.1. Reads were then processed like ChIP-seq reads for mapping with Bowtie2 2.3.4.3 to reference genome mm10 and further processing. Peaks were called with MACS2 2.1.1.20160309 [38] and parameters --no-model, --shift 100, --extsize 200, --keep-dup all and q-value -q 0.01 for pooled replicates because of variable number of replicates.

#### RNA-seq data

RNA-seq data was processed based on [18]: Illumina adapters were trimmed with Trim Galore 0.6.1 [30] and expected FPKM (Fragments Per Kilobase Million) values at gene level were computed with RSEM 1.3.1 [40].

#### Hi-C data

Time series in situ Hi-C data was generated using MboI enzyme and each sample was sequenced to ∼4 billion reads achieving a resolution of 10 kb. The Hi-C data was processed into loop calls based on [41] and as described in detail in [28]. Briefly, read pairs were aligned separately using BWA-MEM [42] to human reference genome hg19, chimeric reads were cut to 5′ ends, low mapping quality filtered, paired and PCR duplicates removed. Juicer [43] tools were used to generate Knight-Ruiz (KR) normalized matrices at 10 kb resolution. Each pixel was compared to its donut region to model expected counts (based on [44]). Candidate pixels were defined as pixels with p-value *<* 0*.*01 and distance *<* 10 kb. Candidate pixels without neigboring candidate pixels were removed. In the last step candidate pixels within 20 kb of each other were collapsed and filtered for p-value *<* 1e-5 to get chromatin loops. Here we employ the candidate pixels before the last collapsing step, which represent significantly in-teracting uncollapsed Hi-C bins of 10 kb.

### Overview of framework

**Fig. 11:**
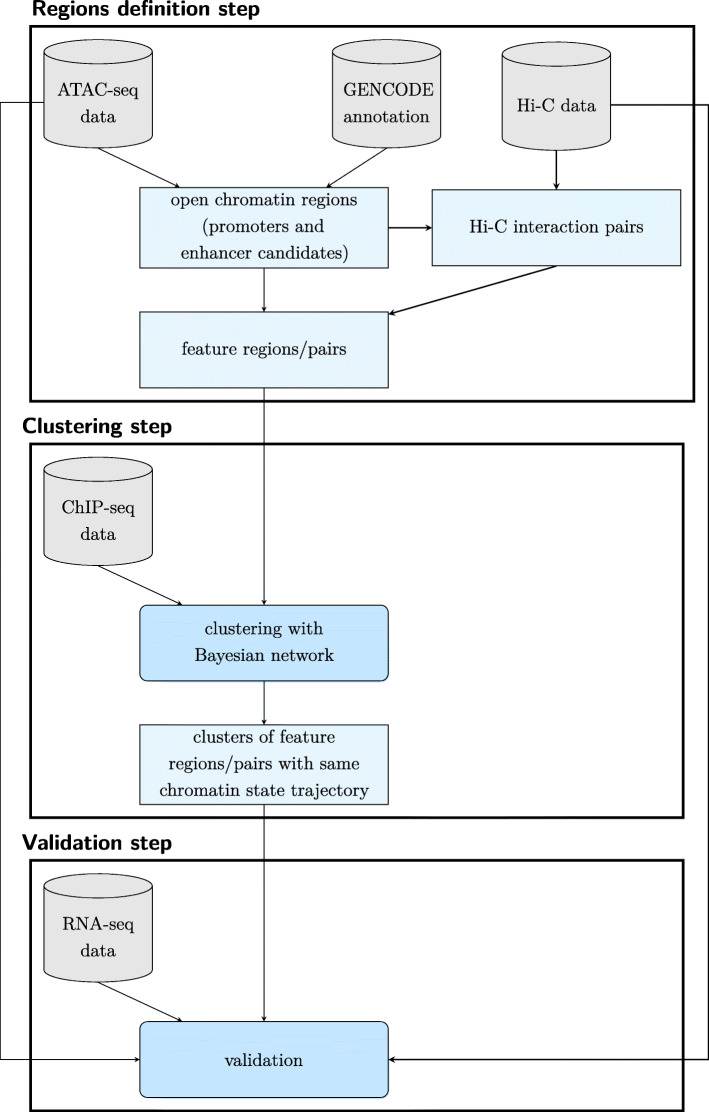
Overview of steps and employed data types in TimelessFlex. The framework TimelessFlex consists of 3 steps (regions definition, clustering and validation step) in which multiple data types are employed

TimelessFlex is a flexible framework for investigating chromatin state trajectories at feature regions around promoters and enhancers or at pairs of such feature regions. TimelessFlex extends Timeless [18] by integrating the additional data types ATAC-seq and Hi-C. The framework can make use of genomic data from multiple biological assays but it is flexible regarding which genomic data is available. An overview of the steps in TimelessFlex and the employed genomic data types is given in Fig. 11. The basic requirements are ChIP-seq data for one or more histone modifications from at least three time points and a set of regions of interest. For the latter, we here use time series ATAC-seq data to define promoters and enhancer candidates, which are partially assigned to promoter-enhancer pairs based on detected Hi-C interactions. These pairs are jointly clustered into paired chromatin state trajectories with an adapted Bayesian network.

### Regions definition step

In this step, promoters and enhancer candidates are defined based on time series ATAC-seq data and assigned to promoter-enhancer pairs based on Hi-C interactions if available. It is recommended to compute the pairs as described in the following, but it is also possible to use pre-computed regions or pairs.

#### Combining ATAC-seq peaks over time into one set of open chromatin regions

For defining promoters and enhancer candidates, we employ time series ATAC-seq data. This is based on the assumption that relevant regulatory regions will be detectable as accessible chromatin regions in at least one time point during a differentiation process that they are functionally active in. Therefore, the sets of ATAC-seq peaks from each time point are combined and then merged if they overlap with a minimal length of 101 bp. This number was choosen as peaks have a minimal length of 200 bp and transitive overlaps should be avoided. Merged regions overlapping ENCODE blacklist regions (mm10 version 2 for mouse data or hg19 original version 1 for human data, both from [45]) were discarded. The resulting merged regions are one final representative set of regions over all time points.

#### Categorization into promoters and enhancer candidates

The total set of open chromatin regions were loosely categorized into promoters and enhancer candidates based on their overlap with GENCODE annotation [46], specifically all transcript TSSs. For mouse data we use GENCODE release M24 and for human data GENCODE release version 29 lift 37 (v29lift37). We use the terms promoters and enhancers as proxies for proximal (TSS adjacent) and distal candidates of regulatory regions. Promoters are defined as regions where: 1) on one strand there is an overlapping TSS and on the other strand there is not, or 2) on both strands there are overlapping TSSs (and the TSS on the plus strand must have a larger coordinate than the TSS on the minus strand). Enhancer candidates are defined as regions where there is no overlapping TSS on either strand.

Regions overlapping multiple TSSs from the same gene are kept and the closest TSS is taken. Overlapping regions and regions that do not fall into any of the categories above, for example overlapping TSSs from multiple genes on the same strand, are discarded. This results in a set of non-overlapping open regions categorized as either promoters or enhancer candidates.

#### Defining feature regions from open chromatin regions

For each candidate regulatory region, we define feature regions as windows around the margins of open chromatin. The feature regions are intended to span the upstream and downstream nucleosomes flanking the open regions. Due to merging the ATAC-seq peaks across multiple samples/time points, the resulting open regions can be quite large (in our case for pancreatic data median lengths of 1200–1400 bp for promoter regions and around 750 bp for enhancer regions). Therefore we chose only 500 bp as extension around the open regions, which ensures that at least the first 2–3 flanking nucleosomes in both directions are captured. To ensure a distinctive signal for the clustering, overlapping feature regions are discarded.

#### Assigning promoters and enhancers to Hi-C interaction pairs

The resolution and coverage of Hi-C data and ATAC-seq data is very different. The resolution of Hi-C data is limited by the enzymatic reaction and sequencing depth, and the highest resolution is currently in the range of 10 kb. Furthermore, it cannot be expected that even deeply sequenced libraries will cover all interactions between regulatory regions. ATAC-seq has in principle single-nucleotide resolution, where it is used for TF footprinting, and the open chromatin regions as derived here have a median width of 700–1400 bp. The candidate assignment of promoters and enhancers to each other was based on Hi-C derived interactions from all time points combined, regardless of the specific time(s) the interaction was detected. When we look at overlaps between Hi-C bins and open chromatin regions, the following cases can occur:

1. Hi-C bin overlaps exactly one open chromatin region fully
2. Hi-C bin overlaps multiple open chromatin regions fully
3. Hi-C bin overlaps only partly or not at all with an open chromatin region

**Fig. 12:**
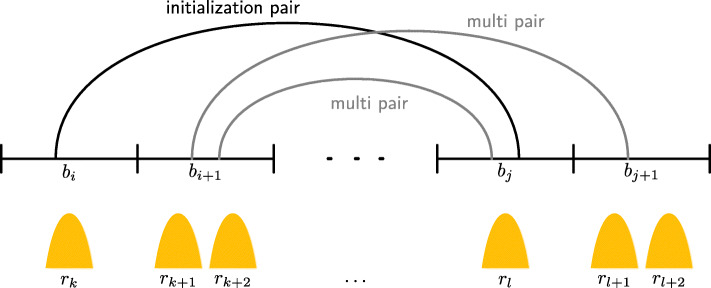
Schematic of initialization and multi pairs from Hi-C data. Initialization (black) and multi (gray) pairs are shown with Hi-C bins *b*_*s*_ and open chromatin regions *r*_*t*_ (yellow). Assignment of pairs leads to initialization pair (*b*_*i*_, *b*_*j*_) and multi pairs (*b*_*i*__*+*__*1*_, *b*_*j*_) and (*b*_i__+__1_, *b*_*j*__*+*__*1*_)

Hi-C bins from case 1 and 2 were used to define Hi-C interaction pairs, and bins from case 3 were discarded. Interaction pairs for which both bins overlap exactly one open regions fully (both bins from case 1) are considered unambiguous and therefore taken as **initialization pairs**. Feature regions around initialization pairs are called initialization feature pairs. Pairs for which one or both bins overlap more than one open region (one or both bins from case 2) are considered ambiguous and referred to as **multi pairs** and their corresponding feature regions as multi feature pairs. Figure 12 illustrates how initialization and multi pairs are defined. Interactions can occur between any type of open chromatin regions; an initialization pair where one bin is overlapping a promoter and one bin is overlapping an enhancer candidate is a promoter-enhancer initialization pair, and analogously for a promoter-enhancer multi pair.

### Clustering step

In this step, histone modification signals at the feature regions are computed, and their normalized changes are used as observable variables in a Bayesian network. The output of this step are clusters of regions with the same chromatin state trajectories.

#### Computing histone modification signals over feature regions

To compute histone modification signals over feature regions, the Signal Generator routine from JAMM [34] was used (following the previous model Timeless [18]). Signal Generator was run for each histone mark and each time point with feature regions (−r), bin size of 1 (−b 1) and depth normalization (−n depth). The values for the parameter -f are the fragment lengths that are computed by JAMM during peak calling and stored in the output file. The output of Signal Generator is a depth-normalized bedgraph file, and for each feature region the maximum signal across the region was taken (see Fig. 2, bottom). Then for each histone modification, the maximum signals were quantile normalized [47] over time to allow comparisons between data sets. Finally, for each histone modification, log2-fold changes between neighboring timepoints were computed and these relative changes were used as input for the clustering.

#### Clustering of histone modification signals with Bayesian network

The log2-fold changes between histone modifications of consecutive time points are clustered with a Bayesian network adapted from [18]. The Bayesian network defines a joint probability distribution over the random variables *C* and ${X}_t^h$, where *C* ∈ {1*, ..., N*} is a hidden discrete random variable that represents the cluster IDs of chromatin state trajectories and ${X}_t^h$ is an observed univariate conditional linear Gaussian random variable which stores normalized log2-fold changes of histone modification *h* at time interval *t*.

The Bayesian network represents the following probability distribution:

$$ {\displaystyle \begin{array}{l}p\left(C,{X}_1^1,\dots, {X}_T^1,{X}_1^2,\dots, {X}_T^2,\dots, {X}_1^H,\dots, {X}_T^H\right)\\ {}=p\left(C\left|{X}_{\mathrm{pa}(C)}\right.\right)\cdot p\;\left({X}_1^1\left|{X}_{\mathrm{pa}\left({X}_1^1\right)}\right.\right)\cdots p\left({X}_T^H\left|{X}_{\mathrm{pa}\left({X}_T^H\right)}\right.\right)\\ {}=p\;(C)\cdot \prod \limits_{h=1}^H\prod \limits_{t=1}^T\;p\;\left({X}_t^h\left|{X}_{\mathrm{pa}\left({X}_t^h\right)}\right.\right),\end{array}} $$

where *X*_pa(*Y*_) are parents of *Y* .

**Fig. 13:**
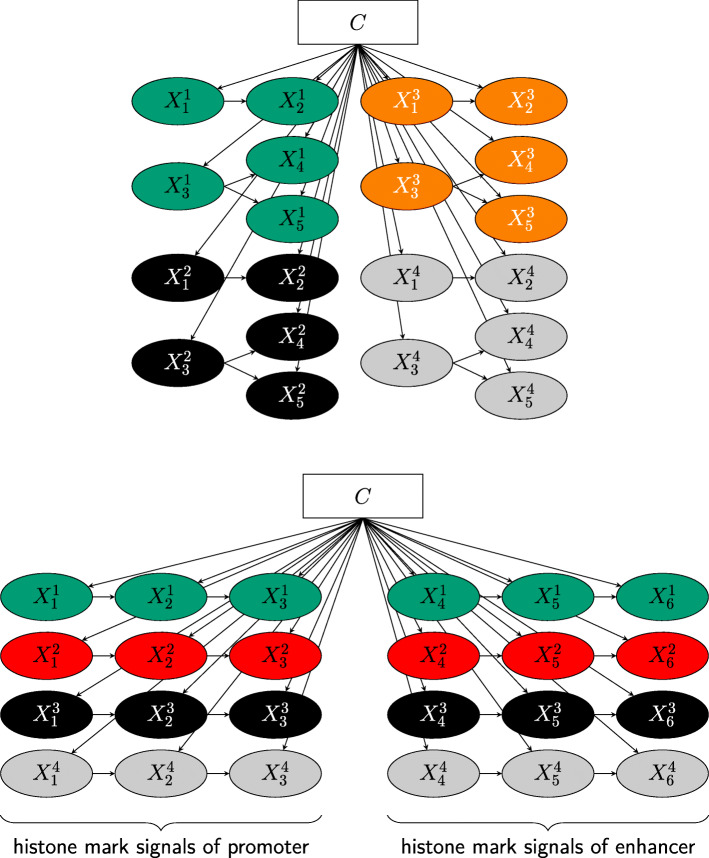
DAGs of Bayesian network for clustering feature regions (top) and promoter-enhancer feature pairs (bottom). Colors represent different histone modifications (green: H3K27ac, red: H3K27me3, black: H3K4me1, orange: H3K4me2, gray: H3K4me3). For clustering of feature regions from mouse hematopoiesis data (top) there are 4 histone modifications and 5 time intervals, therefore the DAG consists of 21 nodes. For clustering of promoter-enhancer feature pairs from human pancreatic differentiation (bottom) there are 4 histone modifications and 3 time intervals resulting in 25 nodes. One half of the continuous nodes represents histone mark signals of the promoter side and the other half represents histone mark signals of the enhancer side

The directed acyclic graphs (DAGs) of the Bayesian network and the random variables for clustering feature regions from mouse hematopoiesis data and promoter-enhancer feature pairs from human pancreatic differentiation are shown in Fig. 13. For time intervals *t* = 1*, ..., T* and histone modifications *h* = 1*, ..., H*, the number of nodes in the TimelessFlex DAG for clustering feature regions is (*H* × *T)* + 1. For clustering feature pairs there are (2 × *H* × *T)* + 1 nodes in the TimelessFlex DAG because it contains a set of promoter-enhancer feature pairs for histone modifications, so that one side represents the histone mark signals of the promoter side and the other side the histone mark signals of the enhancer side.

Without Hi-C data, the feature regions are first filtered to keep only those with a histone mark signal above mean signal on at least one time point. As the cluster assignment is unobservable, the parameters of the model cannot be computed directly. We use *k*-means as initialization to learn model parameters via the EM algorithm. For inference, the junction tree algorithm is used. For each feature region, this results in the likelihoods that a region is assigned to a cluster *C* ∈ {1*, ..., N*} given the fold changes of histone modifications. The cluster with the highest probability is used as the cluster assignment of the region.

When there is an accompanying Hi-C data set available, a two-step strategy is applied: in a first step, the initialization feature pairs, i. e. those with unambiguous promoter-enhancer Hi-C assignments, are clustered. The initialization feature pairs are divided into signal and noise pairs, where signal pairs are pairs with histone mark signals above the mean signal on both feature regions. Then, (*k* − 1)-means is used for the signal pairs, and the noise pairs are assigned to noise cluster *k* as initialization for the EM algorithm. Cluster assignments of feature pairs are generated as described above.

In the next step, the multi feature regions are clustered. The model resulting from the initialization pairs is used to generate cluster assignments as initialization for the EM algorithm. The cluster number is fixed and cluster assignments are computed as described.

#### Model selection

To decide on the number of clusters, we use Bayesian information criterion (BIC) and Akaike information criterion (AIC) computed as follows:

$$ \mathrm{AIC}=-2\cdot \log (L)+2\cdot k $$

$$ \mathrm{BIC}=-2\cdot \log (L)+\log (N)\cdot k $$

where *L* is likelihood of the model, *N* is number of observations (data points) and *k* is degrees of freedom (number of parameters).

For visualization of the resulting clusters, normalized counts are used. Each histone modification is scaled between 0 and 100, and the mean values with error bars are shown.

### Validation step

#### Validation of clusters with genomic data not used in clustering

The resulting clusters of chromatin state trajectories were validated by available genomic data that was not used for clustering itself, for example time series RNA-seq, ATAC-seq or Hi-C data. Note that ATAC-seq is only used to define the coordinates of candidate regions, and Hi-C only to determine promoter-enhancer pairs – i. e., in both cases, neither time point nor quantitative values influence the clustering. The goal was to see if these data support the inferred chromatin state trajectory patterns.

To check how the pancreatic promoter clusters correlate with gene expression, RSEM [40] was used to calculate expected FPKM values from the accompanying time series RNA-seq data set. As a gene can have multiple TSSs and therefore multiple promoter feature regions, it can happen that these regions get assigned to different clusters. We only take genes into account that are assigned to exactly one cluster. For each time point in a cluster, the logarithm of the geometric average of the expected FPKMs plus 1 was finally computed.

To see how accessibility changes over time in the clusters, the time series ATAC-seq signal representing the cut sites over the clustered feature regions is computed. Normalized 1 bp bedgraphs of ATAC-seq data are used, and for each time point, the length normalized number of cut sites in each region was determined. For each cluster, quantile normalization of these data over time was used to allow for comparisons across time. Resulting ATAC-seq signals were normalized and divided by 2.

As assigning promoter-enhancer pairs via Hi-C did not take the time point of the interaction into account, the clustering does not use information at which time point interactions occurred. Hi-C interaction signals between promoter-enhancer pairs visualize how many interactions are present at which time point. The number of interactions between pairs is determined for each cluster and time point, and normalized by the overall number of Hi-C interactions that occur at this time point for all the pairs in the clusters.

All results are visualized with the R barplot or box plot function (outliers are not depicted).

#### Functional interpretation of clusters

##### Promoter clusters

Lists of pancreatic stage-specific signature genes based on gene expression were reported in [21]: 685 genes for D2, 155 for D5 and 236 for D10. We only use the subset of those genes that are in the Hi-C pairs of the clustering. For promoter clusters, the overlap with these genes is computed, but only genes that could be unambiguously assigned during the clustering are taken into account.

##### Enhancer clusters

To find enriched known motifs in the clusters of enhancer candidates, HOMER suite (v4.10) [48] with the script findMotifsGenome.pl was used. It was run for a given cluster using the enhancers as input, the masked genome (-mask), the given region sizes (-size given) and all enhancers from all clusters as background (-bg regions_all.txt).

Intersections between pancreatic clusters of enhancers and published ChIP-seq peaks for FOXA1, FOXA2 and PDX1 (from [20]) were investigated. After liftOver of peaks to hg19 coordinates, pairwise intersections were computed with Intervene [49] followed by Fisher’s two-tailed test to see if the amount of overlap is more than expected given their coverage and genome size.

### Implementation

Wrappers are implemented mostly in bash and R [50]. The framework uses GNU core utilities and multiple publicly available bioinformatics software tools. For clustering, the Bayes Net Toolbox (BNT) in MATLAB [51] is used (Matlab R2016a (9.0.0.341360) 64-bit (glnxa64), February 11, 2016). The pipeline is intended to run on a high performance computing cluster. To enable reproducibility a GNU Guix profile [52] was generated and a Docker [53] image of the profile is available for download at https://bimsbstatic.mdc-berlin.de/ohler/henriettemiko/TimelessFlex-docker-pack.tar. This image contains all packages needed for the framework except for Matlab for the clustering and the packages intervene and csvkit for intersections with published ChIP-seq peaks. All code can be found at: https://github.com/henriettemiko/TimelessFlex.

## Availability and requirements

Project name: TimelessFlex

Project home page: https://github.com/henriettemiko/TimelessFlex

Operating system(s): Linux

Programming language: bash, R, Matlab

Other requirements: Bayes Net Toolbox (BNT) in Matlab, GNU core utilities, multiple publicly available bioinformatics software tools

License: GNU GPLv3

Any restrictions to use by non-academics: Matlab license needed

## Supplementary Information

**Additional file 1: Figure S1.** Model selection for clustering of enhancer feature regions during mouse hematopoiesis. Bayesian information criterion (BIC) and Akaike information criterion (AIC) are computed in the range of 2 to 30 clusters to decide on the number of clusters. Cluster number 19 is a local minimum in the investigated range and was chosen as cluster number.

**Additional file 2: Figure S2.** All 19 clusters of enhancer feature regions during mouse hematopoiesis. Chromatin state trajectories are shown for each cluster.

**Additional file 3: Figure S3.** Model selection for clustering of enhancer feature regions during human pancreatic differentiation. Bayesian information criterion (BIC) and Akaike information criterion (AIC) are computed in the range of 2 to 30 clusters to decide on the number of clusters. Cluster number 8 is a local minimum in the investigated range and was chosen as cluster number.

**Additional file 4: Figure S4.** All 8 clusters of enhancer feature regions during human pancreatic differentiation. Chromatin state trajectories are shown for each cluster.

**Additional file 5: Figure S5.** All 10 clusters of initialization promoter-enhancer feature pairs during human pancreatic differentiation. Chromatin state trajectories and gene expression signals from RNA-seq are shown for each cluster.

**Additional file 6: Figure S6.** Comparison of gene expression signals for closest TSS and Hi-C supported genes. For all 10 clusters of initialization promoter-enhancer feature pairs the gene expression signal from RNA-seq for genes with closest TSSs to enhancers (left) and for Hi-C supported assigned genes (right) is shown.

**Additional file 7: Figure S7.** All 10 clusters of multi promoter-enhancer feature pairs during human pancreatic differentiation. Only the selected multi regions are plotted. Chromatin state trajectories and gene expression signals from RNA-seq are shown for each cluster.

## Data Availability

ATAC-seq and ChIP-seq data for mouse hematopoietic differentiation were downloaded from GEO under accession number GSE59636 [19]. ATAC-seq data for human pancreatic differentiation have been deposited in GEO under accession number GSE151769. ChIP-seq data for H3K27ac and H3K4me1 were downloaded from GEO under accession number GSE54471 [20], ChIP-seq data for H3K27me3 and H3K4me3 were downloaded from Array Express under accession number E-MTAB-1086 [21] and from GEO under accession number GSE149148 [28]. RNA-seq data were downloaded from Array Express under accession number E-MTAB-1086 [21]. Hi-C data were downloaded from the 4D Nucleome Data Portal [29] under accession numbers 4DNESOLVRKBM, 4DNESOL9JVE2, 4DNESV11RYSF and DNESSSDVO27.

## Abbreviations

AIC: Akaike information criterion
BIC: Bayesian information criterion
CMP: Common myeloid progenitor
DAG: Directed acyclic graph
DE: Definitive endoderm
EM: Expectation-maximization
ENCODE: Encyclopedia of DNA Elements
EryA: Erythrocyte A
ES: Embryonic stem
FPKM: Fragments Per Kilobase Million
GMP: Granulocyte macrophage progenitor
Granu: Granulocyte
GT: Gut tube
HMM: Hidden Markov Model
H3K27ac: H3 lysine 27 acetylation
H3K27me3: H3 lysine 27 trimethylation
H3K4me1/2/3: H3 lysine 4 mono−/di−/trimethylation
IDR: Irreproducible Discovery Rate
KR: Knight-Ruiz
LT-HSC: Long-term hematopoietic stem cell
MEP: Megakaryocyte erythroid progenitor
Mono: Monocyte
PE: Pancreatic endoderm
TF: Transcription factor
