# Supplementary figures and images for: Inferring time series chromatin states for promoter-enhancer pairs based on Hi-C data
Source: BMC Genomics. 2021 Jan 28;22:84. doi: 10.1186/s12864-021-07373-z (PMC7841892; doi:10.1186/s12864-021-07373-z)

Bayesian and Akaike information criterion for model selection

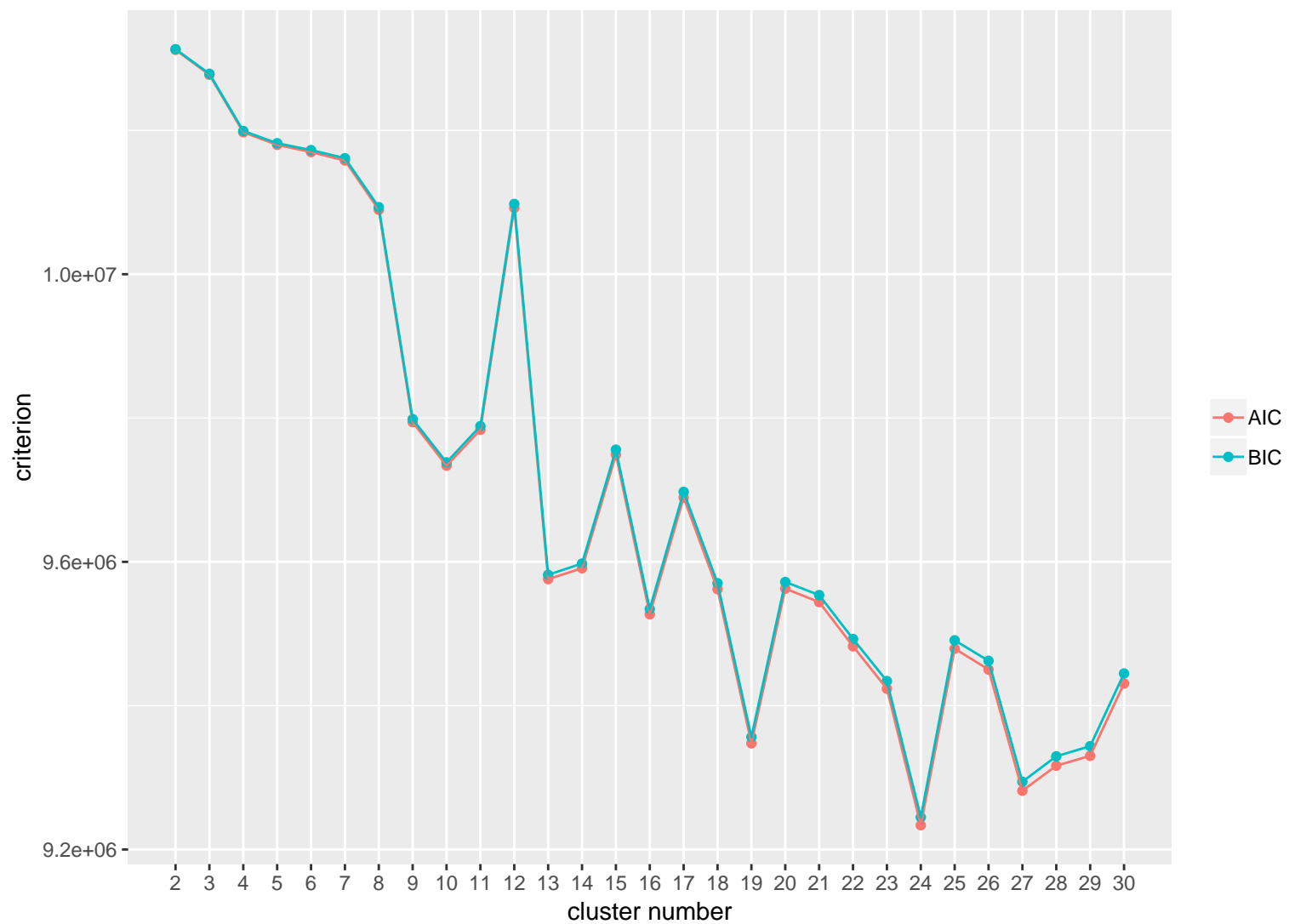

Supplement: Supplementary file 1 — Additional file 1: Figure S1. Model selection for clustering of enhancer feature regions during mouse hematopoiesis. Bayesian information criterion (BIC) and Akaike information criterion (AIC) are computed in the range of 2 to 30 clusters to decide on the number of clusters. Cluster number 19 is a local minimum in the investigated range and was chosen as cluster number. [file 12864_2021_7373_MOESM1_ESM.pdf]

Bayesian and Akaike information criterion for model selection

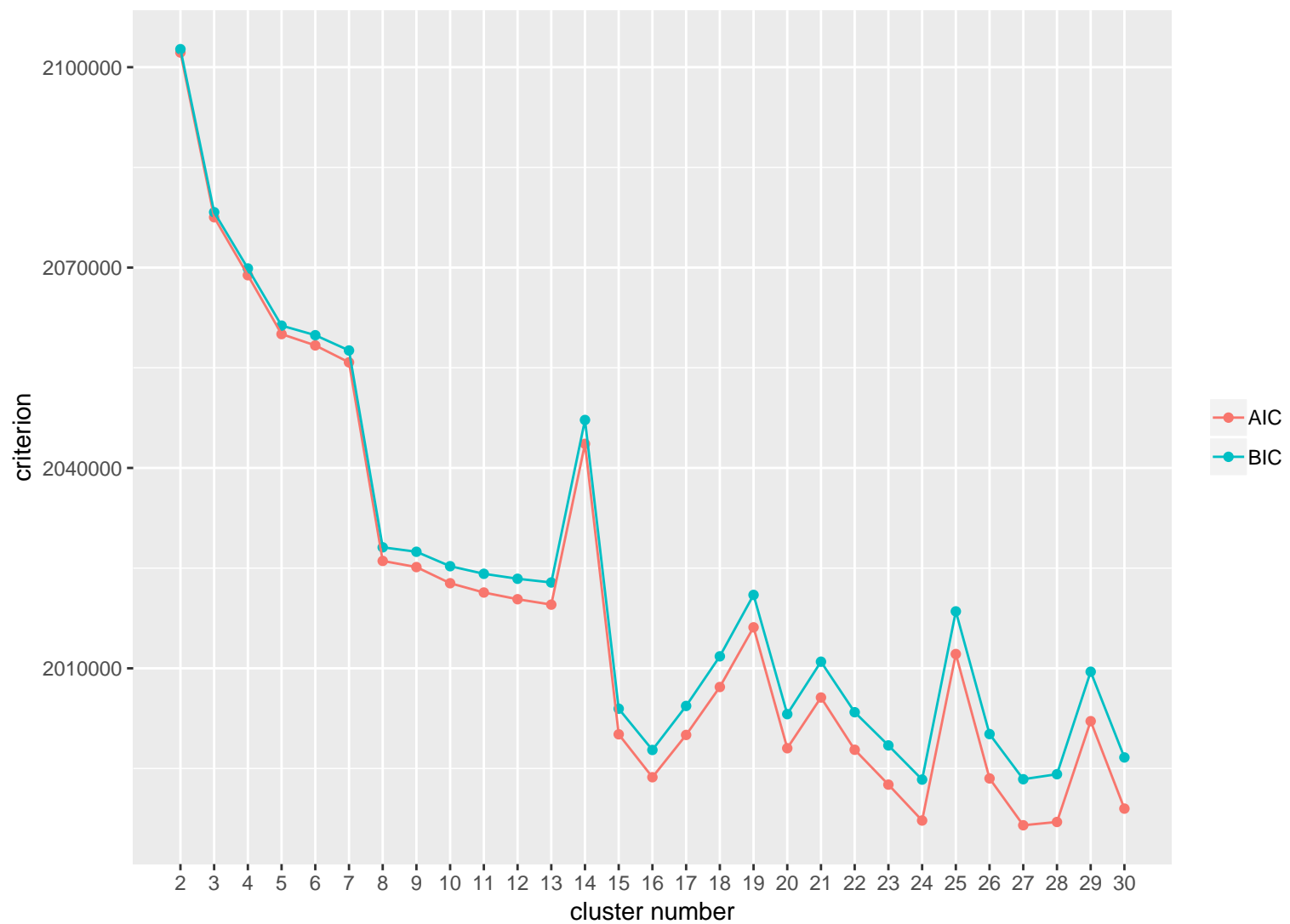

Supplement: Supplementary file 3 — Additional file 3: Figure S3. Model selection for clustering of enhancer feature regions during human pancreatic differentiation. Bayesian information criterion (BIC) and Akaike information criterion (AIC) are computed in the range of 2 to 30 clusters to decide on the number of clusters. Cluster number 8 is a local minimum in the investigated range and was chosen as cluster number. [file 12864_2021_7373_MOESM3_ESM.pdf]

### Cluster 1

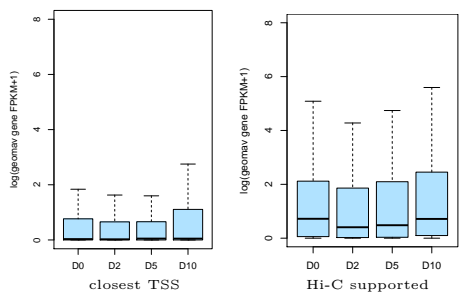

### Cluster 6

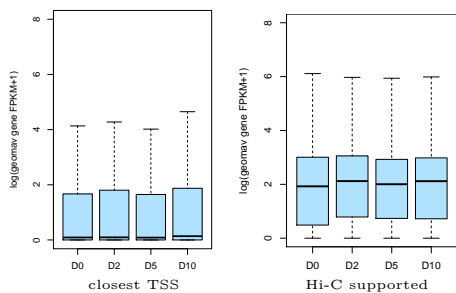

## Cluster 2

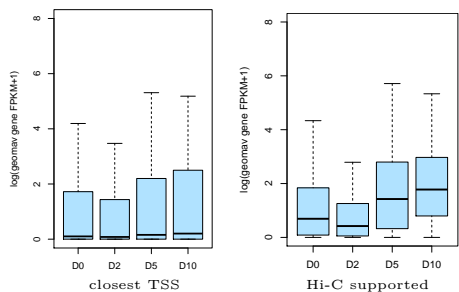

### Cluster 7

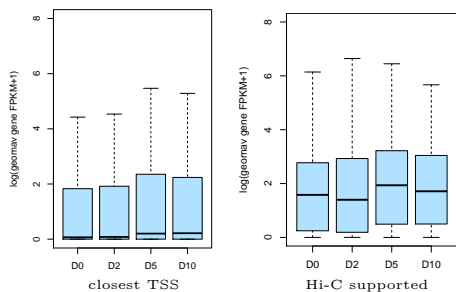

### Cluster 3

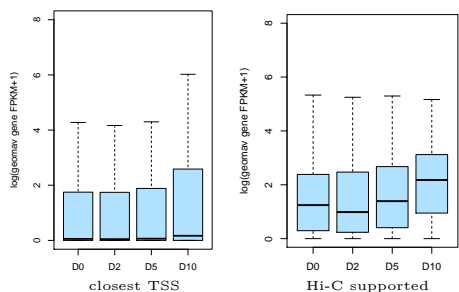

Cluster 8

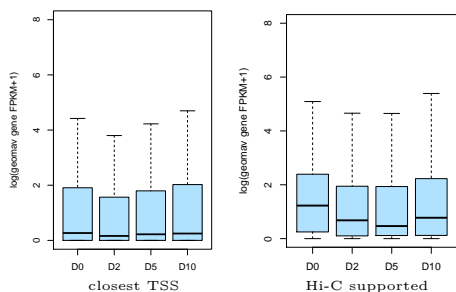

### Cluster 4

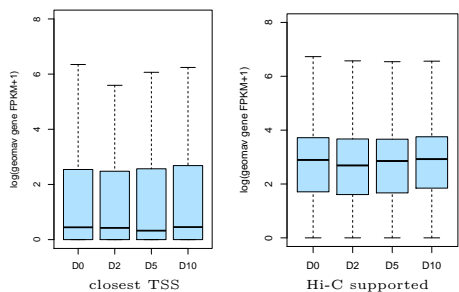

### Cluster 9

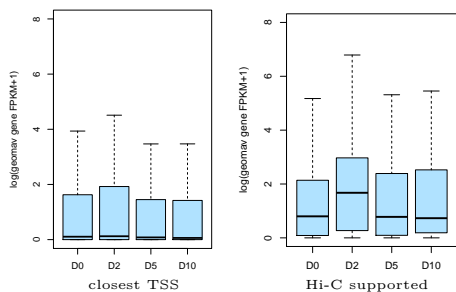

### Cluster 5

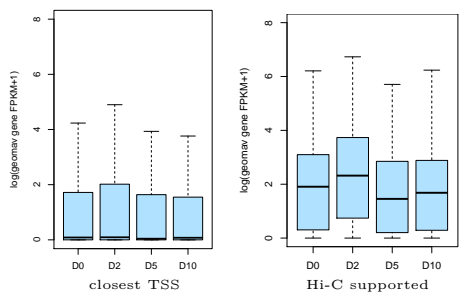

Cluster 10

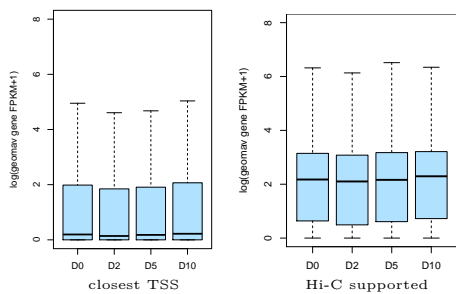

Supplement: Supplementary file 6 — Additional file 6: Figure S6. Comparison of gene expression signals for closest TSS and Hi-C supported genes. For all 10 clusters of initialization promoter-enhancer feature pairs the gene expression signal from RNA-seq for genes with closest TSSs to enhancers (left) and for Hi-C supported assigned genes (right) is shown. [file 12864_2021_7373_MOESM6_ESM.pdf]
