# Supplementary material for: Inferring time series chromatin states for promoter-enhancer pairs based on Hi-C data
Source: BMC Genomics. 2021 Jan 28;22:84. doi: 10.1186/s12864-021-07373-z (PMC7841892; doi:10.1186/s12864-021-07373-z)

Cluster 1
(3583 feature regions)

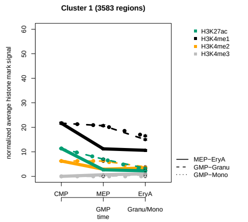

Cluster 2
(1116 feature regions)

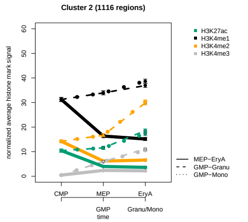

Cluster 3
(355 feature regions)

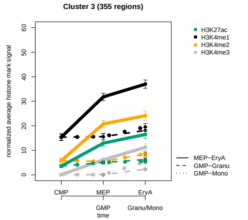

Cluster 4
(1307 feature regions)

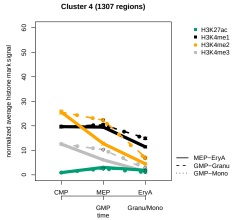

Cluster 5
(11880 feature regions)

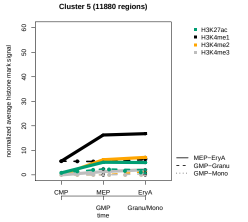

Cluster 6
(1374 feature regions)

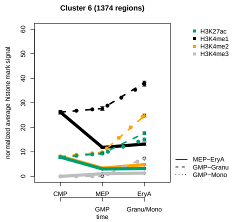

Cluster 7
(983 feature regions)

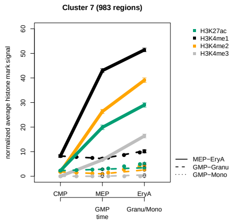

Cluster 8
(993 feature regions)

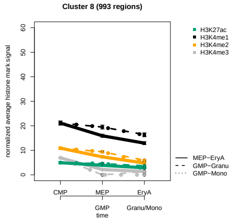

Cluster 9
(977 feature regions)

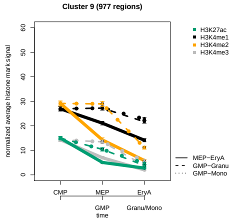

Cluster 10
(1311 feature regions)

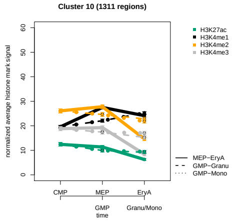

Cluster 11
(2480 feature regions)

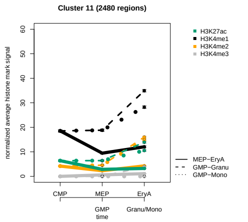

Cluster 12
(3228 feature regions

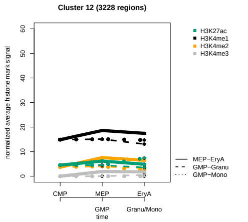

Cluster 13
(4134 feature regions)

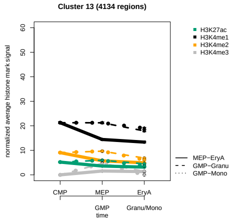

Cluster 14
(1335 feature regions)

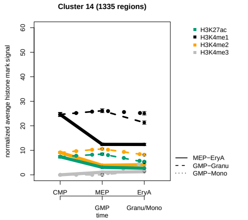

Cluster 15
(727 feature regions)

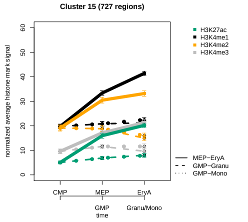

Cluster 16
(7676 feature regions)

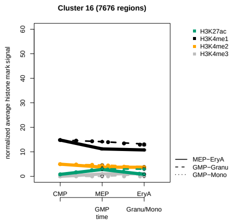

Cluster 17
(1362 feature regions)

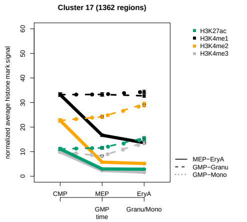

Cluster 18
(933 feature regios)

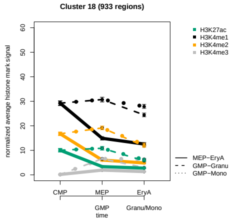

Cluster 19
(3050 feature regions)

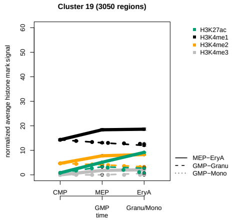

Supplement: Supplementary file 2 — Additional file 2: Figure S2. All 19 clusters of enhancer feature regions during mouse hematopoiesis. Chromatin state trajectories are shown for each cluster. [file 12864_2021_7373_MOESM2_ESM.pdf]
