# Supplementary material for: Inferring time series chromatin states for promoter-enhancer pairs based on Hi-C data
Source: BMC Genomics. 2021 Jan 28;22:84. doi: 10.1186/s12864-021-07373-z (PMC7841892; doi:10.1186/s12864-021-07373-z)
Supplement: Supplementary file 4 — Additional file 4: Figure S4. All 8 clusters of enhancer feature regions during human pancreatic differentiation. Chromatin state trajectories are shown for each cluster. [file 12864_2021_7373_MOESM4_ESM.pdf]

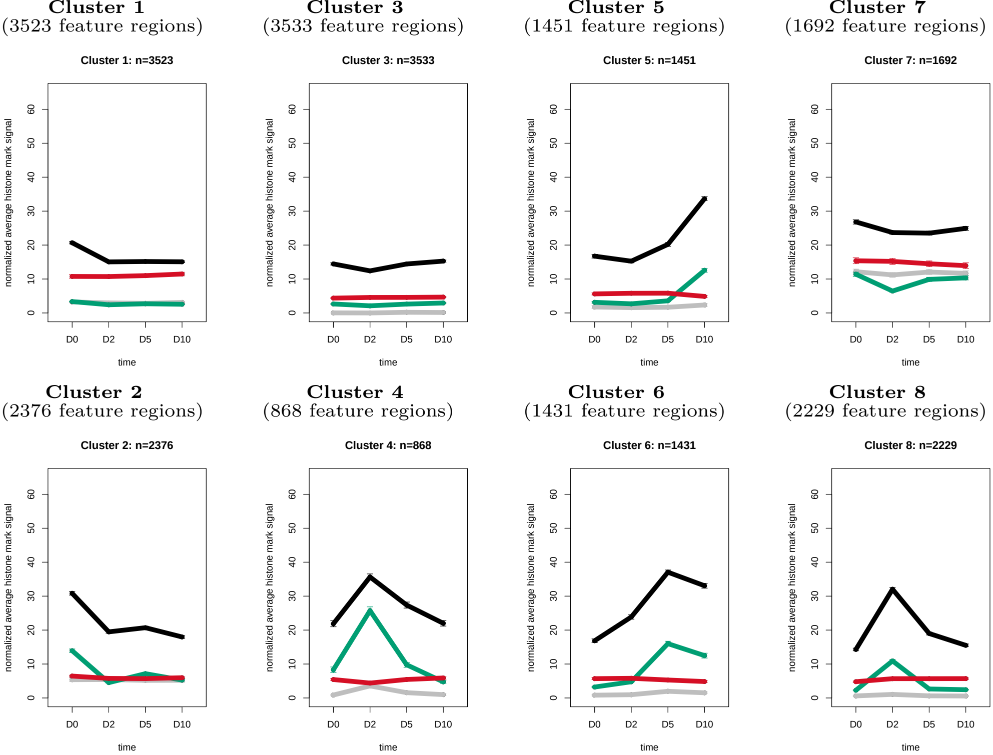
Cluster 1
(3523 feature regions)
Cluster 1: n=3523
Cluster 3
(3533 feature regions)
Cluster 3: n=3533
Cluster 5
(1451 feature regions)
Cluster 5: n=1451
Cluster 7
(1692 feature regions)
Cluster 7: n=1692
Cluster 2
(2376 feature regions)
Cluster 2: n=2376
Cluster 4
(868 feature regions)
Cluster 4: n=868
Cluster 6
(1431 feature regions)
Cluster 6: n=1431
Cluster 8
(2229 feature regions)
Cluster 8: n=2229
normalized average histone mark signal
0
10
20
30
40
50
60
D0
D2
D5
D10
time
