# Supplementary material for: Inferring time series chromatin states for promoter-enhancer pairs based on Hi-C data
Source: BMC Genomics. 2021 Jan 28;22:84. doi: 10.1186/s12864-021-07373-z (PMC7841892; doi:10.1186/s12864-021-07373-z)

Cluster 1 (245 initialization feature pairs)

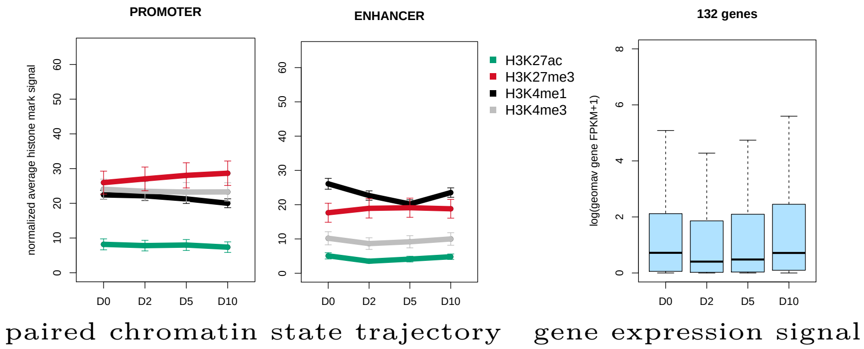

Cluster 2 (211 initialization feature pairs)

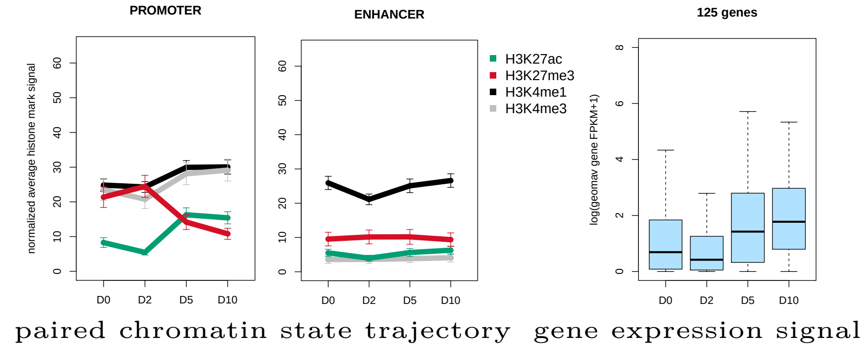

Cluster 3 (282 initialization feature pairs)

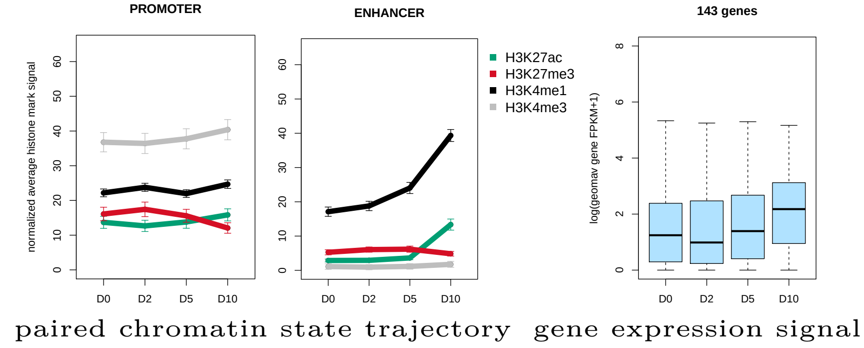

Cluster 4 (597 initialization feature pairs)

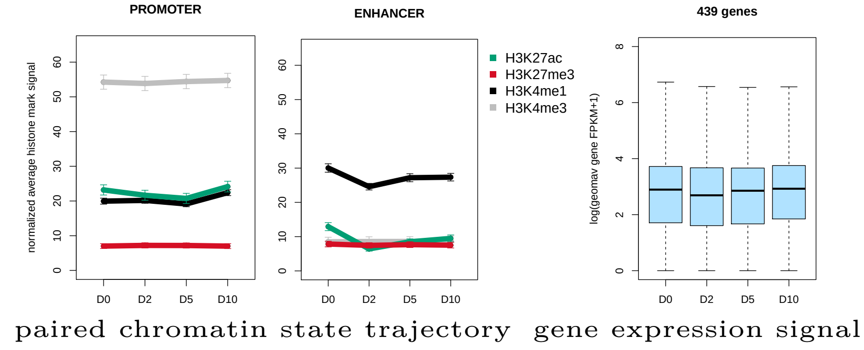

Cluster 5 (151 initialization feature pairs)

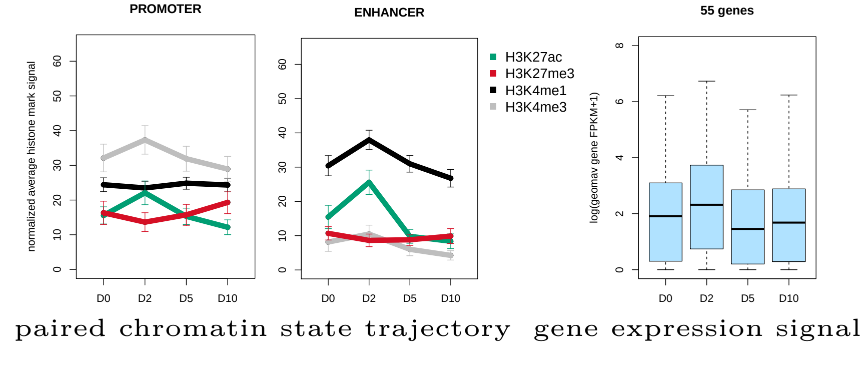

Cluster 6 (367 initialization feature pairs)

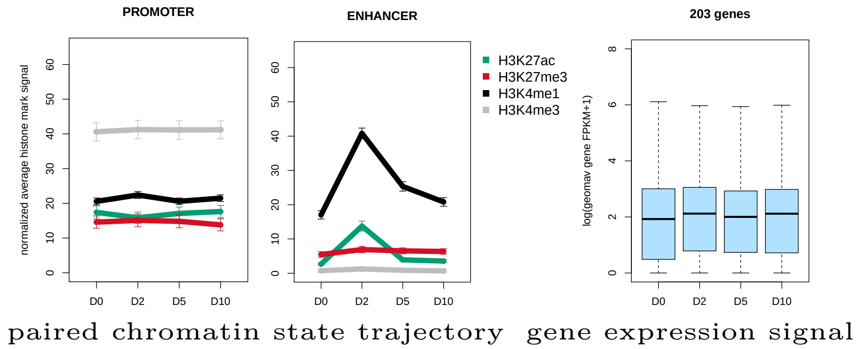

Cluster 7 (226 initialization feature pairs)

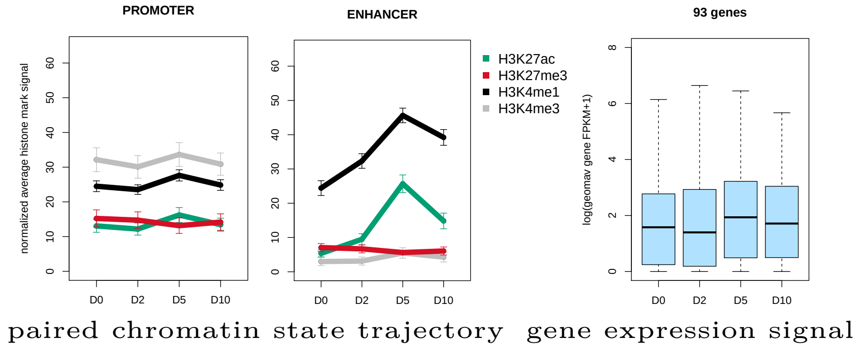

Cluster 8 (358 initialization feature pairs)

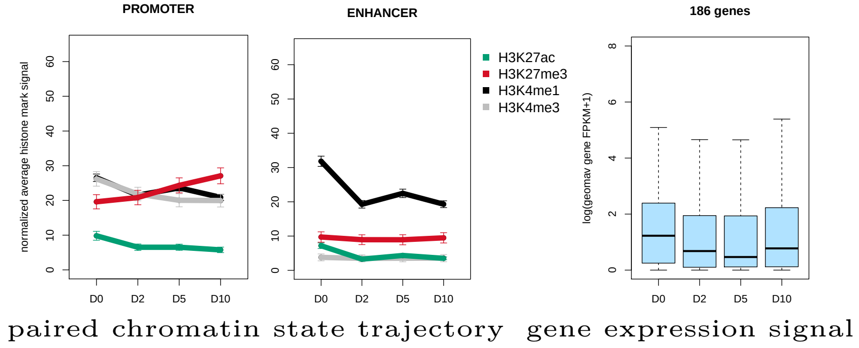

Cluster 9 (286 initialization feature pairs)

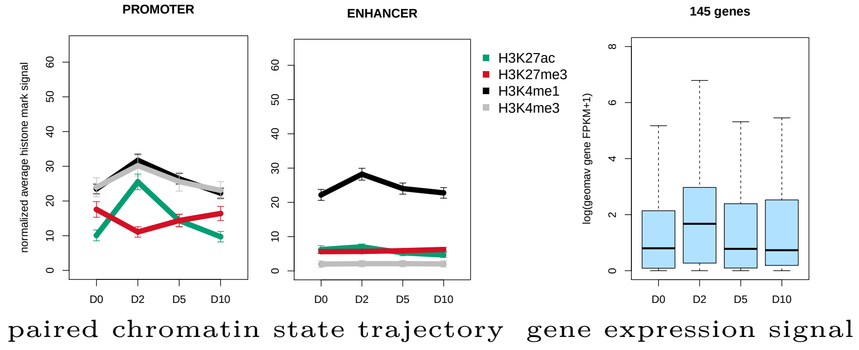

Cluster 10 (894 initialization feature pairs)

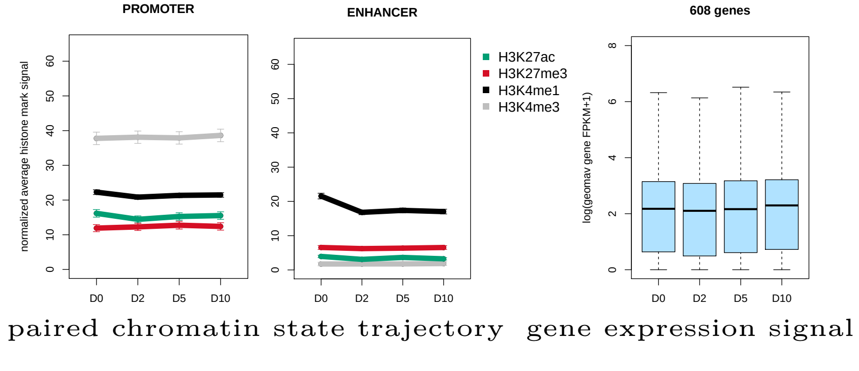

Supplement: Supplementary file 5 — Additional file 5: Figure S5. All 10 clusters of initialization promoter-enhancer feature pairs during human pancreatic differentiation. Chromatin state trajectories and gene expression signals from RNA-seq are shown for each cluster. [file 12864_2021_7373_MOESM5_ESM.pdf]
