# Supplementary material for: Inferring time series chromatin states for promoter-enhancer pairs based on Hi-C data
Source: BMC Genomics. 2021 Jan 28;22:84. doi: 10.1186/s12864-021-07373-z (PMC7841892; doi:10.1186/s12864-021-07373-z)
Supplement: Supplementary file 7 — Additional file 7: Figure S7. All 10 clusters of multi promoter-enhancer feature pairs during human pancreatic differentiation. Only the selected multi regions are plotted. Chromatin state trajectories and gene expression signals from RNA-seq are shown for each cluster. [file 12864_2021_7373_MOESM7_ESM.pdf]

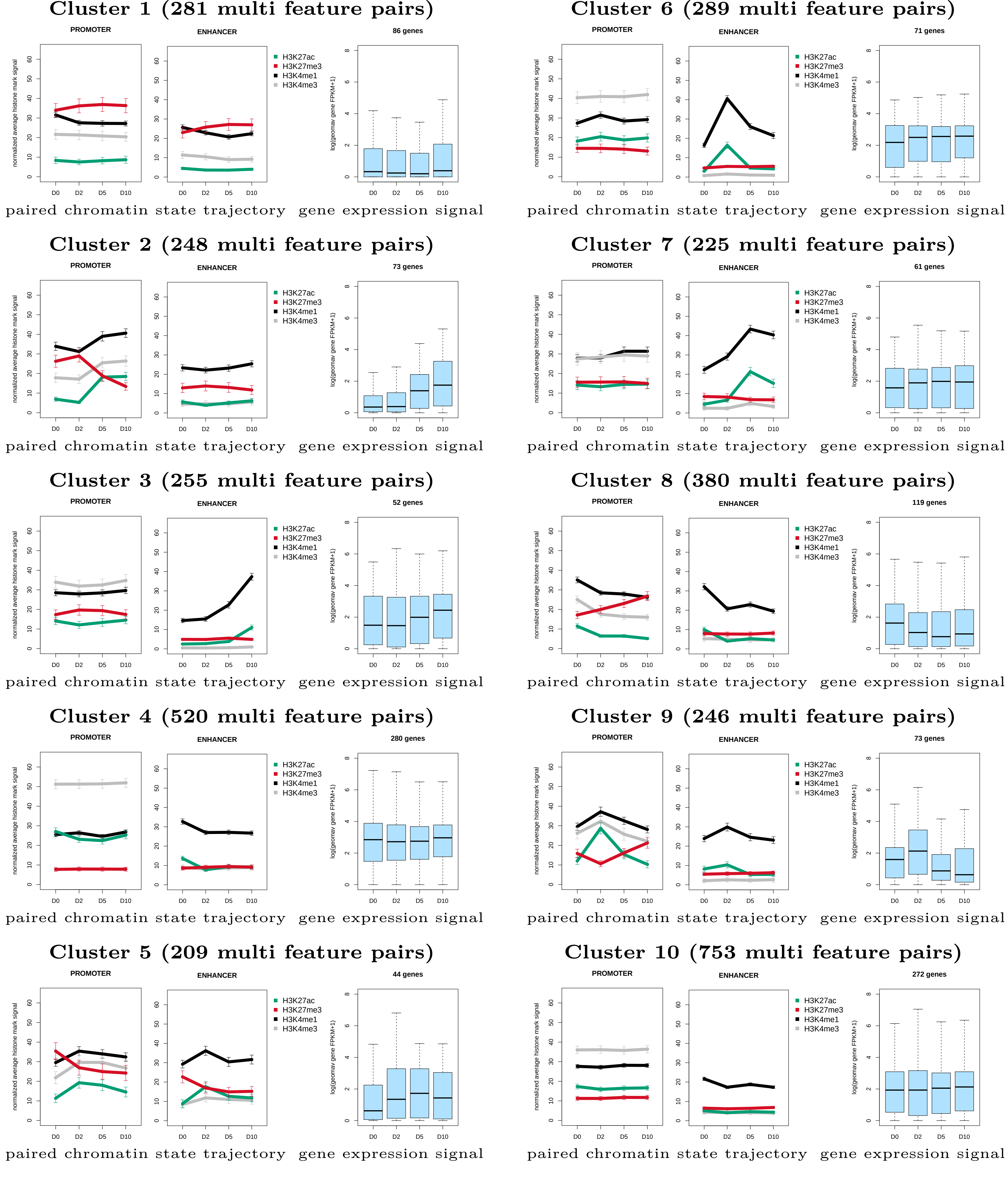

Cluster 1 (281 multi feature pairs)
PROMOTER
ENHANCER
86 genes
H3K27ac
H3K27me3
H3K4me1
H3K4me3
normalized average histone mark signal
log(geomav gene FPKM+1)
D0
D2
D5
D10
paired chromatin state trajectory
gene expression signal
Cluster 2 (248 multi feature pairs)
PROMOTER
ENHANCER
73 genes
paired chromatin state trajectory
gene expression signal
Cluster 3 (255 multi feature pairs)
PROMOTER
ENHANCER
52 genes
paired chromatin state trajectory
gene expression signal
Cluster 4 (520 multi feature pairs)
PROMOTER
ENHANCER
280 genes
paired chromatin state trajectory
gene expression signal
Cluster 5 (209 multi feature pairs)
PROMOTER
ENHANCER
44 genes
paired chromatin state trajectory
gene expression signal
Cluster 6 (289 multi feature pairs)
PROMOTER
ENHANCER
71 genes
paired chromatin state trajectory
gene expression signal
Cluster 7 (225 multi feature pairs)
PROMOTER
ENHANCER
61 genes
paired chromatin state trajectory
gene expression signal
Cluster 8 (380 multi feature pairs)
PROMOTER
ENHANCER
119 genes
paired chromatin state trajectory
gene expression signal
Cluster 9 (246 multi feature pairs)
PROMOTER
ENHANCER
73 genes
paired chromatin state trajectory
gene expression signal
Cluster 10 (753 multi feature pairs)
PROMOTER
ENHANCER
272 genes
paired chromatin state trajectory
gene expression signal
